# Novel disease syndromes unveiled by integrative multiscale network analysis of diseases sharing molecular effectors and comorbidities

**DOI:** 10.1186/s12920-018-0428-9

**Published:** 2018-12-31

**Authors:** Haiquan Li, Jungwei Fan, Francesca Vitali, Joanne Berghout, Dillon Aberasturi, Jianrong Li, Liam Wilson, Wesley Chiu, Minsu Pumarejo, Jiali Han, Colleen Kenost, Pradeep C. Koripella, Nima Pouladi, Dean Billheimer, Edward J. Bedrick, Yves A. Lussier

**Affiliations:** 1 Center for Biomedical Informatics and Biostatistics, The University of Arizona, Tucson, AZ 85721 USA; 2 Department of Medicine at the College of Medicine–Tucson, The University of Arizona, Tucson, AZ 85721 USA; 3 Graduate Interdisciplinary Program in Statistics, The University of Arizona, Tucson, AZ 85721 USA; 4 The Center for Applied Genetics and Genomics Medicine, The University of Arizona, Tucson, AZ 85721 USA; 5 The Center for Innovation in Brain Science, The University of Arizona, Tucson, AZ 85721 USA; 6 UA Cancer Center, The University of Arizona, Tucson, AZ 85721 USA; 7 University of Arizona Health Sciences, The University of Arizona, Tucson, AZ 85721 USA; 8 Epidemiology and Biostatistics Department, College of Public Health, The University of Arizona, Tucson, AZ 85721 USA; 9 Department of Biosystems Engineering, The University of Arizona, Tucson, AZ 85721 USA; 10 Department of Systems & Industrial Engineering, The University of Arizona, Tucson, AZ 85721 USA

**Keywords:** Disease comorbidities, GWAS studies, eQTL, Genetic network, Non-coding variants, RNA, SNP, Diseases, Complex diseases, Intergenic, Common diseases

## Abstract

**Background:**

Forty-two percent of patients experience disease comorbidity, contributing substantially to mortality rates and increased healthcare costs. Yet, the possibility of underlying shared mechanisms for diseases remains not well established, and few studies have confirmed their molecular predictions with clinical datasets.

**Methods:**

In this work, we integrated genome-wide association study (GWAS) associating diseases and single nucleotide polymorphisms (SNPs) with transcript regulatory activity from expression quantitative trait loci (eQTL). This allowed novel mechanistic insights for noncoding and intergenic regions. We then analyzed pairs of SNPs across diseases to identify shared molecular effectors robust to multiple test correction (False Discovery Rate FDR_eRNA_ < 0.05). We hypothesized that disease pairs found to be molecularly convergent would also be significantly overrepresented among comorbidities in clinical datasets. To assess our hypothesis, we used clinical claims datasets from the Healthcare Cost and Utilization Project (HCUP) and calculated significant disease comorbidities (FDR_comorbidity_ < 0.05). We finally verified if disease pairs resulting molecularly convergent were also statistically comorbid more than by chance using the Fisher’s Exact Test.

**Results:**

Our approach integrates: (i) 6175 SNPs associated with 238 diseases from ~ 1000 GWAS, (ii) eQTL associations from 19 tissues, and (iii) claims data for 35 million patients from HCUP. Logistic regression (controlled for age, gender, and race) identified comorbidities in HCUP, while enrichment analyses identified cis- and trans-eQTL downstream effectors of GWAS-identified variants. Among ~ 16,000 combinations of diseases, 398 disease-pairs were prioritized by both convergent eQTL-genetics (RNA overlap enrichment, FDR_eRNA_ < 0.05) and clinical comorbidities (OR > 1.5, FDR_comorbidity_ < 0.05). Case studies of comorbidities illustrate specific convergent noncoding regulatory elements. An intergenic architecture of disease comorbidity was unveiled due to GWAS and eQTL-derived convergent mechanisms between distinct diseases being overrepresented among observed comorbidities in clinical datasets (OR = 8.6, *p*-value = 6.4 × 10^− 5^ FET).

**Conclusions:**

These comorbid diseases with convergent eQTL genetic mechanisms suggest clinical syndromes. While it took over a decade to confirm the genetic underpinning of the metabolic syndrome, this study is likely highlighting hundreds of new ones. Further, this knowledge may improve the clinical management of comorbidities with precision and shed light on novel approaches of drug repositioning or SNP-guided precision molecular therapy inclusive of intergenic risks.

**Electronic supplementary material:**

The online version of this article (10.1186/s12920-018-0428-9) contains supplementary material, which is available to authorized users.

## Background

Comorbidity, or the co-occurrence of two or more diseases with each other, is a widespread phenomenon with estimates suggesting that 42% of all patients have at least one comorbidity [1]. For example, comorbid metabolic syndrome, presenting at least two diseases from a cluster of metabolic underlying medical conditions, afflicts 47 million people in the US alone [2]. Comorbidities do not occur randomly, and the excess observation of specific disease-disease co-occurrence in clinical records can imply shared underlying pathophysiological mechanisms [3]. Another recent study showed that 120 disease-trait pairs (e.g., acute lymphoblastic leukemia-mean corpuscular volume) with shared genetic architecture using curated gene association studies significantly co-occurred in electronic health records [4].

Big data science approaches have begun to characterize biomolecular mechanisms that lead to cross-disease relationships; however, these have primarily focused on specific disease-disease hypotheses or required biochemically well-described disease-protein associations as input (e.g., using protein-protein interactions [5, 6]). In addition, very few studies have validated their predictions of comorbid syndromes (a group of medical conditions consistently occurring together) by observation in clinical datasets [7]. Specifically, these few computational biology studies have shown to correlate with clinical comorbidity: (i) the presence of shared gene expression and flux coupling in metabolic pathways of disease-causing genes [8], (ii) the overlap of disease-associated host genes of polymorphisms and their interacting proteins or functional annotations [9, 10], (iii) the comorbidity of diseases sharing Mendelian genetics [11], (iv) the overrepresentation of Mendelian disease genes in differentially expressed genes of cancers [12], and (v) the genetic or phenotypic network proximity observed in databases of complex and Mendelian genetics [13]. *However, all of these approaches focused on candidates within the protein-coding genome, with intergenic regions neglected by design*. Yet, intergenic and noncoding regions of the genome contain the majority of genetic variants associated with common diseases according to genome-wide association studies (**GWAS**) [7, 14]. Despite this abundance of noncoding genetic signals, the role of these intergenic polymorphisms in the pathology of clinical comorbidities remains insufficiently characterized [15, 16].

*We hypothesize that intergenic variants explain in part the emergence of comorbidities through their role in gene regulation.* Variants located far from coding regions and noncoding variants could cause comorbidity by regulating expression levels of messenger RNA transcripts, which are associated with both diseases. These regulated genes can presumably be imputed using expression quantitative trait loci (**eQTL**) that associate single nucleotide polymorphisms (**SNPs**) with altered levels of messenger RNAs, microRNAs, and/or noncoding RNA transcript expression. Note, we will refer eQTL-associated RNAs as eQTL RNA hereafter. Statistically-significant overlap of eQTL-associated RNAs whose expression is regulated by distinct disease-associated SNPs could identify a novel shared biomolecular mechanism between a disease-disease pair, or comorbid syndromes in general. This relationship could then be validated by the overrepresentation of that disease-disease pair (comorbidity) in clinical care.

To examine this hypothesis, we integrated summary statistics from the NHGRI-EBI GWAS Catalog of published genome-wide association studies [14] with eQTL calculated by Fagny and colleagues [17] from the Gene-Tissue Expression (**GTEx**) project [18] to investigate the effects of intergenic variants with an emphasis on *trans*-regulation of gene expression. We previously published precursors to this approach which were limited to lymphoblastic cell line and liver tissue-derived eQTL data [19, 20]. In these two studies, we could accurately predict genetic synergy and antagonism between within-disease SNP pairs which were validated by an independent GWAS. This demonstrates that within-disease-SNP pairs, for which a statistical relationship has been predicted between their downstream messenger RNAs are associated by eQTL, were substantially overrepresented among  interacting chromatin elements found in ChIA-PET data drawn from The Encyclopedia of DNA Elements [21]. With that established, the study in this paper now focuses on convergent mechanisms *between diseases* and leverages a large clinical dataset from 35 million patients.

## Methods

**Fig. 1:**
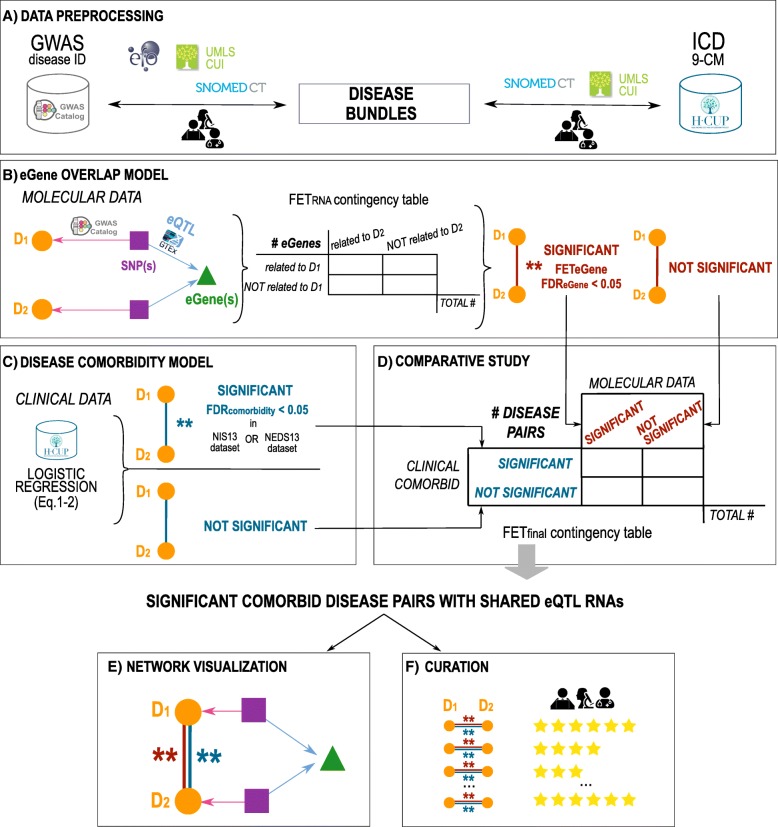
Overview of the study. **a** Preprocessing. We created a controlled disease terminology across the molecular data from the GWAS Catalog and the HCUP clinical datasets (Methods- Data preprocessing to define disease bundles). We mapped GWAS diseases into disease bundles, i.e., group diseases, using the EMBL-EBI EFO, UMLS-CUI, SNOMED-CT nomenclatures integrated with expert curation (Methods- Creation of the SNOMED-coded disease-bundles from GWAS terms). Similarly, we mapped HCUP diseases coded with ICD-9-CM terminology into disease bundles by using SNOMED-CT, UMLS, and expert curation (Methods- Mapping HCUP diseases to disease-bundles). **b** eQTL RNA overlap model. Convergence between downstream eQTLs signals associated with coding and intergenic disease-associated polymorphisms are calculated for each pair of diseases (Methods- Statistical overlap). We selected significant disease pairs sharing convergent mechanisms by applying the Fisher’s Exact Test (FET) according to the contingency table shown in the panel. We considered significant disease pairs surpassing FDR_eRNA_ of 0.05. **c** Disease comorbidity model. We computed the disease comorbidity for each disease pairs by applying logistic regression (Methods- Calculation of disease comorbidity) to the clinical datasets. The effect size and significance of disease co-occurrence in clinical datasets (comorbidities) were controlled for age, gender, and race. Significant comorbid disease pairs were selected accordingly with FDR values (FDRcomorbidity < 0.05). **d** Comparative study. Finally, congruence between molecular-prioritized disease pairs and clinically-prioritized comorbidities is measured by applying FET-based enrichment studies (FET_final_) (Methods- Comparative studies between eQTLs and HCUP). **e** Network visualization. We further investigated in detail the molecular networks of comorbid disease pairs with sharing convergent genetics (eQTL RNAs) (Methods- Network visualization of the comorbidities sharing intergenic genetic risks). **f** Curation. For additional validation, we conducted a systematic curation of the literature using PUBMED and Google Scholar for the comorbidities discovered from HCUP datasets (FDR < 0.05, OR > 3) having convergent eQTL RNAs (Methods- Curation of prioritized comorbidities)

**Fig. 2:**
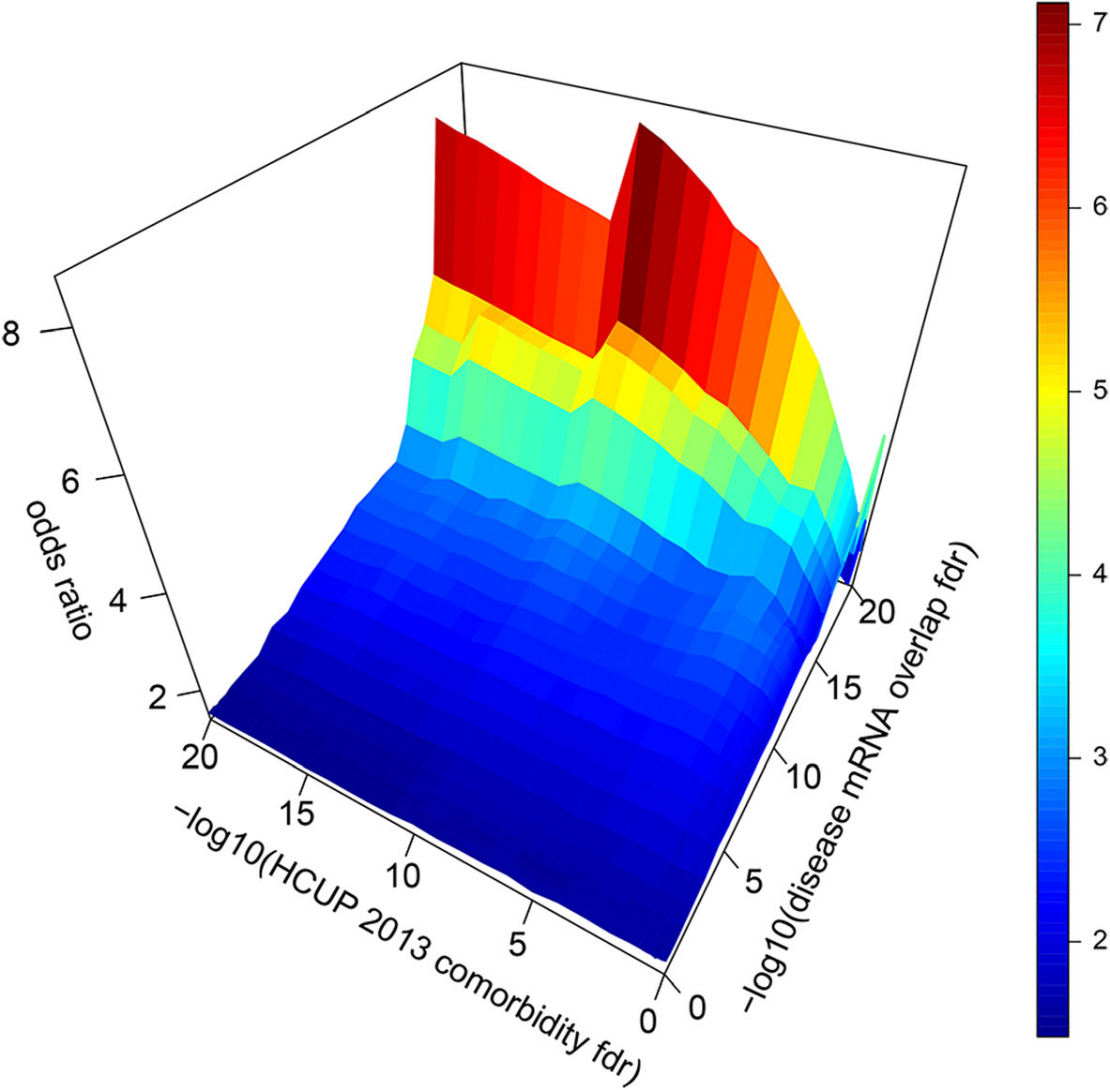
Convergent downstream genetic mechanisms predicted from shared eQTL RNA between disease-pairs are enriched among comorbidities observed in clinical datasets. Vertical axis = odds ratio of overrepresentation of shared molecular mechanisms among clinical comorbidities (Results-Convergent genetic mechanisms between disease-pairs are enriched among comorbid disease). Left bottom axis = FDR cutoffs of comorbidities found in the HCUP clinical datasets (OR > 3; Results- Prioritized comorbidities), right bottom axis = FDR cutoffs of shared molecular mechanisms discovered between two diseases

**Fig. 3:**
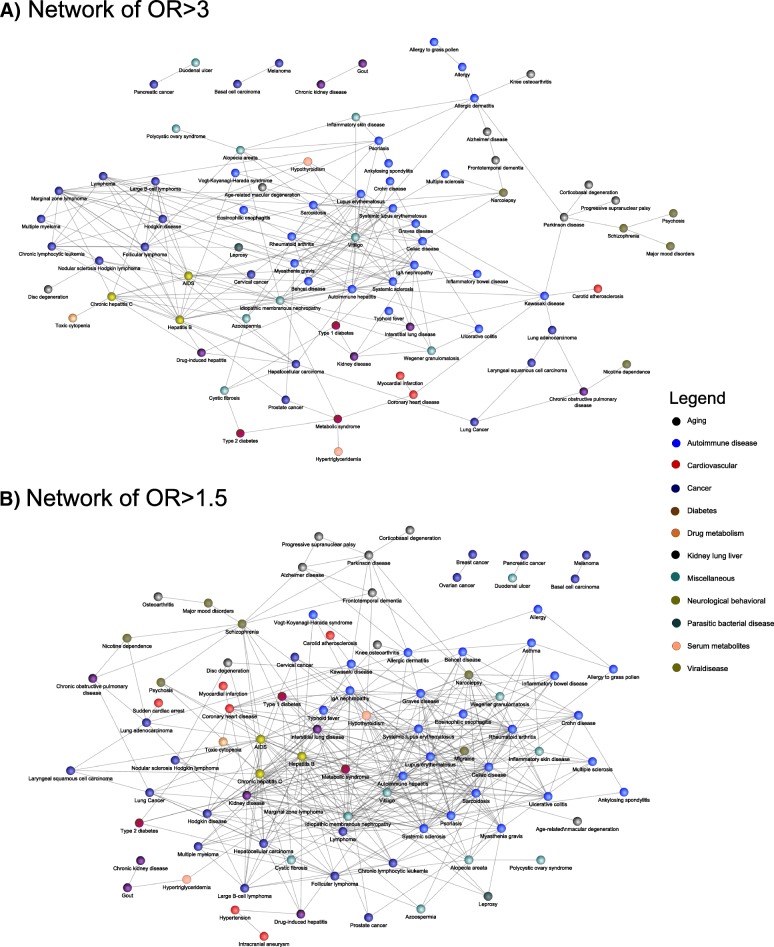
Network of disease-pairs prioritized as comorbid and sharing convergent genetic mechanisms through cis- and trans-eQTL associations of their coding and intergenic polymorphisms. Convergent molecular mechanisms were confirmed at FDR < 0.05 (Methods- Calculation of disease comorbidity based on HCUP). Disease comorbidities were confirmed in either clinical datasets NIS13 or NEDS13 at FDR < 0.05 (Panel **a** with OR > 3; panel **b** with OR > 1.5; Methods- Statistical overlap of eQTL-associated RNAs between distinct disease-associated SNPs). Known clinical syndromes with common genetic risks are recapitulated (e.g., metabolic syndrome), as well as less known monogenic diseases modulated with SNPs unrelated to their monogenic cause (e.g., SNPs worsening cystic fibrosis associated by eQTL studies to those of the metabolic syndrome for which the comorbidity is known but not the underpinning biological mechanisms). Many eQTL mechanisms relate known co-classified diseases (e.g., cancers, immune-mediated diseases), however many cross classes provide intriguing novel comorbidities linked by genetics that had eluded discovery by both clinicians and geneticists (e.g., Parkinson’s disease and Allergic Dermatitis). In most cases, though, the comorbidity was known and explained to clinicians by non-genetic pathophysiology (e.g., duodenal cancer and pancreatic cancer), and yet this study implies that previously undiscovered genetic mechanisms further amplify these comorbid conditions in predisposed individuals. Legend. Edge widths are proportional to the number of tissues that yielded eQTL RNA associations with SNPs by eQTL analyses (19 tissues, eQTL RNA and SNPs not shown; details in Fig. 5 for two examples). Diseases classifications are color-colored (e.g., autoimmune disorders in blue)

**Fig. 4:**
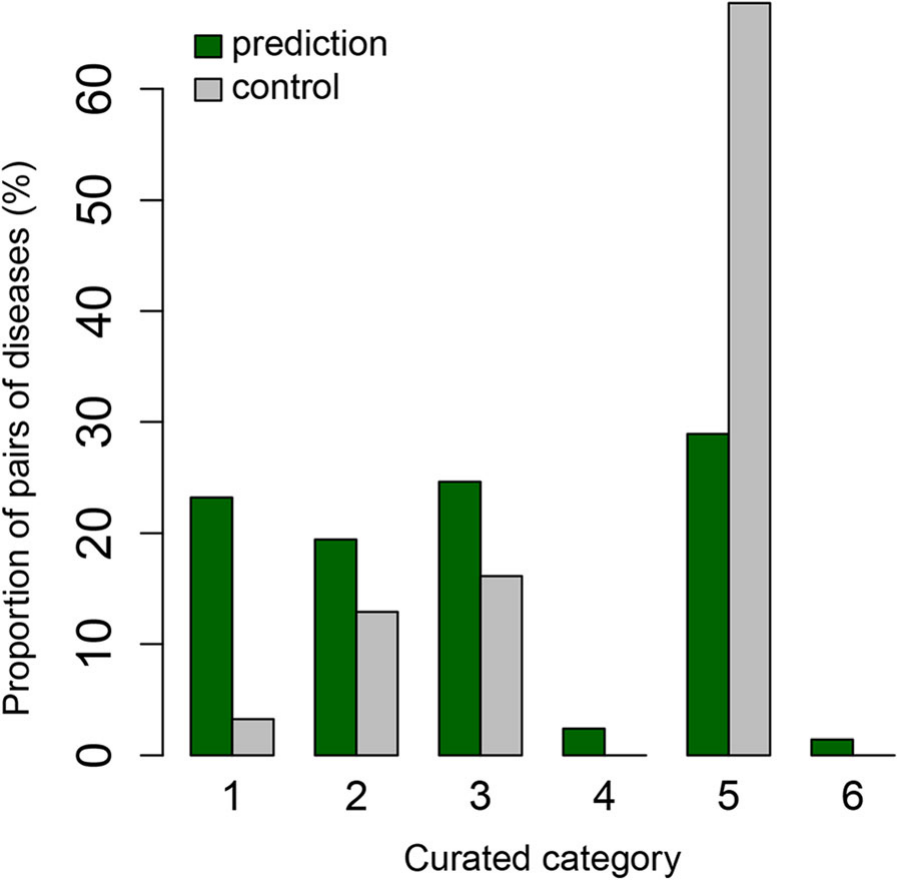
Curation results for prioritized comorbidities with an eQTL downstream convergence. Prioritized comorbidities (green) were enriched in curation categories as compared to blind controls (grey) consisting of random disease pairs among non-prioritized ones (*p* = 0.001; inter-rater agreement *p* < 1.4 × 10^− 3^). Legend: evidence for positive correlation (levels 1–2); no evidence for association or evidence for non-coexistence of diseases (levels 5 or 6), see Methods- Curation of prioritized comorbidities

**Fig. 5:**
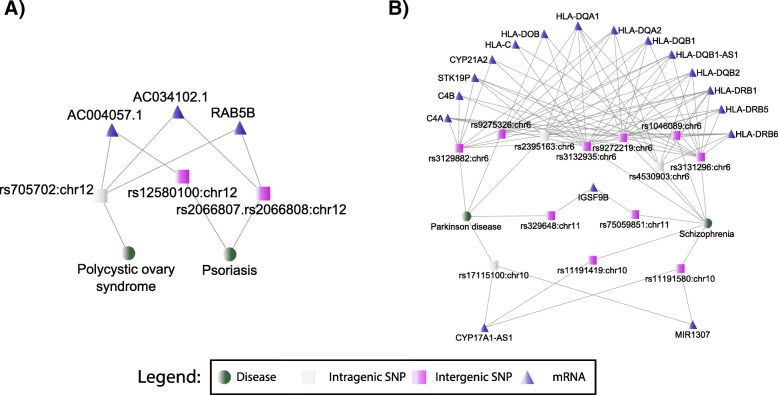
Examples of comorbidities that share downstream intergenic eQTL mechanisms via their associated SNPs. Panel **a**: *Polycystic ovary syndrome* (POS) and *psoriasis* are observed comorbid in HCUP (Odds ratio (OR) = 2.3) and were previously described as co-occurring [45]; however, the common genetic risk remains unreported. Here, we provide evidence that intergenic SNPs of psoriasis share eQTL associations with the intragenic SNPs of POS (LD *r*^2^ = 0.05 on CEU population). Panel **b**: Parkinson’s disease and schizophrenia are also known as comorbid, and here we show a novel shared mechanism among their numerous intergenic SNPs (protein-coding IGSF9B, microRNA MIR1307, and ncRNA CYP17A10-AS1). SNPs on chromosome 11 are 57 k away (LD *r*2 = 0.04), and SNP rs17115100 is 20.9 k byte from rs11191419 (LD *r*2 = 0.15) and 314.8 k byte apart from a LD SNP rs1191580

An overview of this study is shown in Fig. 1 and consists of six major steps: 1) data preprocessing (Fig. 1a), 2) computation of convergent molecular mechanisms between SNP pairs associated with distinct diseases via GWAS and eQTL studies (Fig. 1b), 3) disease comorbidity calculations using Healthcare Cost and Utilization Project (HCUP) clinical datasets (Fig. 1c), 4) comparative study and validation (Fig. 1d), 5) network construction (Fig. 1e), and 6) additional validation through manual curation (Fig. 1f). In detail, the preprocessing step (**Methods- Data preprocessing to define disease bundles and map heterogeneous diseases representation**) consisted of defining the terminology between the disease classes of interest, represented in the GWAS studies, for the purposes of the study, i.e., **disease-bundles**. This disease list was obtained through a semi-automated procedure consisting of expert curation and the use of different data sources, such as the Systematized Nomenclature of Medicine--Clinical Terms (**SNOMED-CT**) [22, 23] and the Unified Medical Language System (**UMLS**) [24]. This also enabled the harmonization of discrepant disease nomenclatures of the GWAS studies and the claim records by defining a controlled disease terminology between the molecular and clinical datasets. The second step included the imputation of molecular convergence between disease bundle pairs and the related statistical significance using eQTL associations of SNPs associated via GWAS to the two diseases in the pair (**Methods- Statistical overlap of eQTL-associated RNAs between distinct disease-associated SNPs**). Next, we used the HCUP Electronic Health Records (**EHR**) datasets to calculate disease comorbidities and their statistical significance (**Methods- Calculation of disease comorbidity based on HCUP**). Finally, to verify our hypothesis, we studied the concordance between the genetic convergence and clinical comorbidity and discuss our findings (**Methods- Comparative studies between eQTLs and HCUP - Curation of prioritized comorbidities**).

### Datasets

The study integrates nine data sources (Table 1). GWAS diseases and their reproducible disease-associated SNPs were downloaded from the **NHGRI-EBI GWAS catalog**. To extract eQTL associations linking SNPs to expressed genes (RNAs), we downloaded files calculated by Fagny and colleagues [17] from the **GTEx** project V6 [18] spanning 19 tissues. The authors determined *cis-* and *trans-*eQTL at *p*-value< 0.2 for 19 tissues. These files were chosen to conduct a more in-depth analysis of trans-eQTLs. For clinical data, we acquired the Healthcare Cost and Utilization Project (**HCUP**) claim datasets. Both the HCUP National Inpatient Sample (**NIS13**) and the HCUP Nationwide Emergency Department Sample (**NEDS13**) datasets were employed to compute and ensure the reproducibility of comorbidities. For the definition of the disease bundles and for mapping purposes, we integrated several phenotype datasets, including EMBL-EBI Experimental Factor Ontology (**EFO**) [14], the **SNOMED-CT**, and the Unified Medical Language System (**UMLS) MRCONSO** file. Finally, other datasets were downloaded as needed for biological study, such as the Single Nucleotide Polymorphism database (**dbSNP**) [25] for intragenic (within gene coordinates) and intergenic (between genes) categorization, and **HapMap** [26], 1000 Genomes Project [27], and **LDlink** [28] for linkage disequilibrium (**LD**) in case studies.

**Table 1:** Data sources

Dataset name	Version	Downloaded	Source (URL)	Data type derived
NIGHRI-EBI GWAS Catalog	2016	07/10/2016	https://www.ebi.ac.uk/gwas/docs/file-downloads	Disease-to-SNP associations derived from GWAS
EMBL-EBI EFO	2016	07/10/2016	https://bioportal.bioontology.org/ontologies/EFO	Disease branches
GTEx	V6	05/04/2017	http://networkmedicine.org:3838/eqtl/	SNP-to-eQTL_RNA relations derived from eQTL studing associating SNPs to the regulated targets (RNAs)
dbSNP	142	08/25/2016	ftp://ftp.ncbi.nlm.nih.gov/snp/organisms/human_9606/ ASN1_flat/	SNP host gene or intergenic SNPs
HCUP	2013	09/01/2016	https://www.distributor.hcup-us.ahrq.gov/	Disease-patient relations
SNOMED	Sep. 2015	11/2015	https://www.nlm.nih.gov/healthit/snomedct/us_edition.html	Disease SNOMED-CT IDs
UMLS	2015AA	07/09/2015	https://www.nlm.nih.gov/research/umls/licensedcontent/umlsarchives04.html	Disease UMLS IDs
HapMap LD	2009	10/11/2010	ftp://ftp.ncbi.nlm.nih.gov/hapmap/ld_data/	Linkage disequilibrium data
1000 Genome	2014	11/14/2014	ftp://ftp.1000genomes.ebi.ac.uk/vol1/ftp/release/20130502/	Linkage disequilibrium r^2

### Data preprocessing to define disease bundles and map heterogeneous diseases representation

The HCUP clinical datasets use ICD-9-CM diagnosis codes while the genetic GWAS dataset uses heterogeneous descriptive language for disease names that vary even in related studies of the same trait. To bridge the heterogeneous disease names between datasets, as a first step (Fig. 1a), we performed semi-automated curation and normalization of the disease names into the SNOMED-CT concepts. The use of SNOMED-CT, i.e., a comprehensive ontology organized as a directed acyclic graph (**DAG**), allowed for the automatic calculation of a semantic relatedness/similarity between the diseases by leveraging the SNOMED-CT hierarchy [23]. Thanks to this procedure, all disease names in the GWAS Catalog were first regrouped into SNOMED-CT classes of proper semantic granularity, i.e., **disease-bundles** (**Methods- Creation of the SNOMED-coded disease-bundles from GWAS terms**). Similarly, each ICD-9-CM code [29] relating to a disease in the HCUP datasets was also mapped to the corresponding SNOMED-CT code, facilitating the comparison with the diseases represented in the GWAS studies (**Methods- Mapping HCUP diseases to disease-bundles**).

### Creation of the SNOMED-coded disease-bundles from GWAS terms

To focus only on diseases from the GWAS Catalog corresponding to disease phenotypes (e.g., removing phenotypes associated with response to therapy or non-disease traits such as skin color), we kept only GWAS traits under the disease branch of the EMBL-EBI EFO, reducing the number of unique traits from 1622 to 533. Next, a first round of curation was performed by physicians, which further reduced these 533 GWAS disease traits to 481 of interest for purposes of the study. Through the disease-EFO mapping, we linked (whenever available) each GWAS disease-trait to external terminology IDs, i.e., SNOMED-CT and ICD-9-CM. To automatically augment the coverage of GWAS trait-to-SNOMED-CT mapping, we first mapped the EFO-linked external IDs to UMLS Concept Unique Identifiers (**CUIs**) using the UMLS MRCONSO table and, second, we obtained the final SNOMED-CT list by retaining all the SNOMED-CT IDs covered under each CUI. After the augmented mapping, we linked 431 disease terms to at least one SNOMED-CT ID.

Next, starting from the resulting mapping, we defined a list of non-redundant and clinically meaningful disease-bundle candidates by applying two criteria: 1) merging pairs of diseases into a disease-bundle if they were mapped to an identical set of SNOMED IDs; and 2) merging pairs of diseases into a disease-bundle if the disease names were identical after removing the ending parenthetical qualifier of the GWAS term, e.g., “Glaucoma” and “Glaucoma (high intraocular pressure)”. For quality control, the disease-bundles underwent iterative curation by a physician, a geneticist, and a clinical informatician. Following this procedure, we finally selected from the GWAS Catalog a total of 238 disease-bundles associated with 6175 SNPs.

Mapping HCUP diseases to disease-bundles (coded in SNOMED-CT).

To compare the disease comorbidity obtained using the HCUP datasets with the disease pairs sharing convergent mechanisms via GWAS and eQTL associations, we had to convert the ICD-9-CM diagnosis codes in the HCUP datasets to the previously identified disease-bundles. Since ICD-9-CM is a classification and SNOMED-CT is a nomenclature, our mapped SNOMED-CT concepts are more comprehensive than ICD-9-CM codes [29]. Therefore, we proceeded by identifying all descendant terms of a SNOMED-CT term associated with a GWAS disease-bundle. Next, the most precise correspondence between SNOMED-CT codes and ICD-9-CM codes were identified using the UMLS dataset. Inconsistent SNOMED-CT-to-ICD-9-CM mappings were detected through a final curation by experts. This procedure enabled the mapping of 2454 ICD-9-CM codes into 188 bundles that occur in the two HCUP datasets.

### Statistical overlap of eQTL-associated RNAs between distinct disease-associated SNPs (eQTL RNA overlap)

To identify the effect size and significance of shared genetic risk mechanisms between two diseases, we assessed the statistical overlap of expressed RNAs associated with disease-associated SNPs by eQTL studies (**eQTL RNA overlap model,** proxy for shared transcript mechanisms). As our focus was specifically on interpretable downstream mechanistic insight through the incorporation of eQTL, we investigated beyond the simple model of SNPs or loci shared by two different diseases (eQTL SNP overlap model). Examples of studies on disease-disease relationships through common shared risk loci (e.g., HLA) can be found in [30–33].

In our model, we integrated disease-to-SNP associations via GWAS with SNP-to-eQTL RNA associations via eQTL. As each disease may have one or many associated SNPs by GWAS and each SNP may regulate the expression of multiple genes via eQTL (eQTL RNAs), acting either in cis or trans, we used SNP-SNP pairs associated with two distinct diseases and with a statistically-significant overlap of RNA(s) to prioritize the disease pairs (Fig. 1b) (described below).

For each SNP-SNP pair where the SNPs are associated with two distinct diseases, we determined whether or not both SNPs were independently associated with regulating the same eQTL RNA transcript within a specific tissue/cell line. Since we focused on SNP-SNP pairs associated to distinct disease pairs, we could determine disease pairs with significant eQTL RNA overlapping using the Fisher’s Exact Test (**FET**_**eRNA**_). In detail, for each of the 19 tissues, we used the FET by taking all expressed eQTL RNAs as background and evaluating whether or not a eQTL RNA was associated with each of the two diseases via the associated SNPs. This allowed us to construct a contingency table (Fig. 1b) and derive a statistical significance (*p*-value) between any pair of diseases for each tissue. *P*-values were adjusted for multiple comparisons by False Discovery Rate (**FDR**_**eRNA**_) using the Benjamini–Hochberg procedure [34].

In our approach, we retained all disease pairs surpassing the stringent cutoff of 0.05 for the FDR values.

### Calculation of disease comorbidity based on HCUP

As mentioned, the comorbidity between bundled diseases from two HCUP datasets, the National Inpatient Sample (NIS13) and the Nationwide Emergency Department Sample (NEDS13), were assessed for robust findings. Two directional comorbidities for every pair of disease-bundles were evaluated separately (Fig. 1c). For each pair of disease-bundles, denoted *D*_*1*_ and *D*_*2*_, we tested the following logistic regression models (*E:* expectation):

1 $$ E\left( logit\left({D}_1\right)\right)={\beta}_{10}+{\beta}_{11}{D}_2+{\beta}_{12} race+{\beta}_{13} sex+{\beta}_{14} age $$

2 $$ E\left( logit\left({D}_2\right)\right)={\beta}_{20}+{\beta}_{21}{D}_1+{\beta}_{22} race+{\beta}_{23} sex+{\beta}_{24} age $$

The two models correspond to the comorbidity risk of *D*_1_ given *D*_2_ and *D*_2_ given *D*_1_ respectively. Covariates (confounders), such as race, sex, and age, were adjusted in both models. ***β***_**ij**_ are logistic coefficients of each variable to be estimated from the data. We chose not to use alternative methods, such as graphical modeling [35] and LASSO [36, 37] since graphical models are typically used to understand the joint dependence structure for a set of variables, while LASSO is commonly used for regularization when there are large numbers of potential effects in the model.

In implementation, we collected the most specific disease codes (all five ICD-9-CM digits) mapped to the disease-bundles. Then, for each patient, we determined whether the patient has a billing code among the mapped ICD-9-CM codes, through which the statuses for a pair of disease-bundles were determined. From all patients without missing required data (e.g., missing sex information), the parameters within the two models were estimated and the significance of the directional comorbidity (***β***_**11**_ and ***β***_**21**_) was inferred. We adjusted multiple comparisons from both models and across all pairs of disease-bundles using Benjamini-Hochberg procedure to derive the False Discovery Rate [34], which derived two directional FDRs for each pair of disease-bundles (e.g., disease A may cause disease B and not the reverse). Disease pairs with FDR < 0.05 in either directional tests were considered as comorbidities. Larger odds ratio (**OR**) were estimated from the power of two coefficients (***β***_**11**_ and ***β***_**21**_) as the OR of comorbidity between each pair of diseases. These calculations were conducted in both HCUP datasets separately and the best *p*-value is reported in Additional file 1 as **FDR**_**comorbidity**_. We further confirmed the ORs from an additional bivariate logistic model within the *Zelig* R package for the retained disease pairs, where the bivariate logistic model is unidirectional and able to estimate the dependence of the two disease variables subjected to covariates, yielding a mean odds ratio between a pair of diseases [38].

### Comparative studies between eQTLs and HCUP

To verify the hypothesis that diseases sharing convergent downstream are more likely to show comorbidities and that an association exists between disease comorbidity and genetic/genomic architectures, we investigated the concordance between disease pairs showing significant downstream eQTL convergence in GWAS (**Methods- Statistical overlap of eQTL-associated RNAs between distinct disease-associated SNPs**) and the pairs of diseases resulted prioritized as comorbid using the HCUP clinical data (**Methods- Calculation of disease comorbidity based on HCUP**). To this end, we performed a FET to test the enrichment of comorbid disease-pairs with the disease pairs sharing eQTL mechanisms (**FET**_**final**_, Fig. 1d). In the FET_final_ test, we examined the number of disease pairs resulted statistically significant (FDR < 0.05)/not significant in the molecular dataset and in the clinical comorbid data. Therefore, the FET_final_ test was conducted by counting the number of disease pairs under each of the four combinative conditions (contingency table shown in Fig. 1d). The signal robustness was verified across different conditions and datasets, different FDR cutoffs (ranging from 0.02 to 1) were evaluated for both comorbidity and eQTL RNA overlap, and the reproducibility and the enrichment trend were examined with respect to the strength of the cutoffs (Fig. 2). The reproducibility from multiple HCUP datasets was also examined using the comorbidity observed in multiple datasets.

In addition, we performed a further validation applying the FET test as previously described but excluded the disease pairs involving similar diseases. This way we could assess the significance of diseases not similar resulted sharing convergent mechanisms as well as comorbid.

To identify similar pairs, we computed a clinical-ontology-based semantic similarity (or distance) between the disease-bundle pairs applying Lin’s similarity metric [39] with Sanchez et al.’s information content estimation [40]. This approach takes into account the SNOMED-CT ontological structure, and SNOMED-CT are used as proxy for phenotypic relatedness of diseases. For each bundle pair, a score between 0 and 1 was derived. We considered two diseases similar if their similarity score was higher than 0.9.

### Network visualization of the comorbidities sharing intergenic genetic risks through eQTL RNA overlap

First, we created a network representing disease pairs (Fig. 1e) including (i) disease pairs sharing genetic mechanism through eQTL RNA overlap of eQTL associations to disease-associated SNPs (**Methods- Statistical overlap of eQTL-associated RNAs between distinct disease-associated SNPs**), and (ii) disease comorbidities in at least one of the two HCUP datasets (**Methods - Calculation of disease comorbidity based on HCUP**). Here, network nodes represent diseases and two nodes (diseases) are linked (network edge) if the related disease pair meet both the above-mentioned comorbidity and eQTL RNA overlap criteria. To every edge, we assigned a weight corresponding to the number of distinct tissues that yielded the significance of the disease pairs. We colored the nodes according to the clinical organ-system classes as defined by Han et al. [19] and adjusted edge width according to the edge-related weight value.

Second, for any interesting overlapping disease pairs, we built a related network to represent the biomolecular mechanisms underlying the comorbidity between the two diseases. Therefore, a network was built for each comorbid pair by connecting each disease in the pair to the related SNPs via GWAS and then associating each SNP to the overlapping RNAs resulted via the eQTL associations. Nodes of the resulting network can represent a disease, a SNP, or a RNA, and edges can correspond to disease-to-SNP or SNP-to-eQTL RNA associations. The biomolecular network of a comorbid disease pair therefore includes the common downstream RNAs (prioritized at FDR < 0.05) between the corresponding prioritized and disease-associated SNPs (**Methods- Calculation of disease comorbidity based on HCUP**). Irrelevant information for the comorbidity (e.g., insignificant eQTL RNA overlap) that can be derived from other edges is not shown. Note, we grouped as a single locus (LD ≥ 0.8) the SNP pairs associated with the same disease and that are in Linkage Disequilibrium (**LD**). All networks were visualized using Cytoscape [41].

### Curation of prioritized comorbidities

For the comorbidities discovered from HCUP datasets (FDR < 0.05, OR > 3) that have convergent eQTL downstream genes, we conducted a systematic curation of the literature using PUBMED and Google Scholar (Fig. 1f). Disease names were searched in PUBMED and Google Scholar and abstracts of the resulted papers were checked for comorbidity evidence. Full texts were examined if a conclusion of comorbidity could not be concluded in the abstracts. For quality control, three independent curators carried out the curation and resolved. In addition, 15% of random disease pairs (controls), selected among pairs with no comorbidity in either HCUP datasets nor convergent eQTL mechanisms in any GTEx tissue, were added to the curation list (blind to curators). An inter-rater agreement was computed using the Spearman correlation test, while disagreement was thereafter solved under the supervision of an expert physician. Next, we categorized the resulting curation evidence of a disease pair into six levels: 1 = well-performed controlled studies confirming the positive comorbidity, 2 = evidence from studies with important limitation (e.g., small sample size), 3 = anecdotal case reports, 4 = strong evidence of absence of association (well-controlled studies, no significance), 5 = absence of studies in the literature, and 6 = strong evidence for an anti-correlation or non-coexistence of the diseases. Finally, to assess our results, we compared the frequencies of levels of evidence confirming the “prioritized comorbidities sharing molecular mechanisms” with those of the random disease-pair controls using the Chi-square test since multiple levels were involved in the test.

## Results

### Preprocessing results

We mapped the diseases extracted from the GWAS Catalog and HCUP datasets into disease bundles as described in **Methods- Data preprocessing to define disease bundles and map heterogeneous diseases representation**. In detail, 262 disease bundles were associated with 429 diseases collected from the GWAS (Additional file 2). Out of these 262 diseases, 188 are associated with SNPs having eQTL associations with at least one eQTL RNA. On the other hand, using the clinical HCUP datasets, we associated 238 disease bundles with 2454 ICD-9-CM diseases (Additional file 3).

### Disease pairs with convergent eQTL-mechanisms of genetic polymorphisms

The eQTL RNA overlap model allowed for the identification of shared RNAs associated with eQTL SNPs that were also significantly associated with two distinct diseases (FET_eQTL_). We conducted the eQTL RNA enrichment among all the possible combinations of the 188 disease bundles associated with SNPs present in at least one eQTL study, using each of the 19 GTEx tissues. The possible disease pairs combinations resulting from the 19 tissues were 16,320 (Note: this number is not the possible theoretical combination between all the 188 diseases since not all the pairs were present in all tissues). Starting from the GTEx studies, for each tissue, we extracted the eQTL SNPs and RNAs associated to the diseases (Table 2; Input columns) and computed the eQTL mRNA overlap model to extract significant disease pairs with convergent molecular mechanisms (**Methods- Statistical overlap of eQTL-associated RNAs between distinct disease-associated SNPs**). Overall, we prioritized 2043 distinct disease pairs with significant eQTL RNA overlap (FDR_eRNA_ < 0.05; across all tissues) out of these possible pairs. In our calculation, we considered SNP-SNP pairs and the related eQTL RNAs where each SNP was associated to a distinct disease. Therefore, we specifically tested for cis- and trans-eQTL overrepresentation from coding as well as noncoding and intergenic SNPs. Note that the number of significant disease pairs varied from tissue to tissue, with lung tissue yielding the minimal number (45) of disease pairs and skin tissue yielding the maximal number (1775) of disease pairs (Table 2; Output column).

**Table 2:** Count of prioritized disease pairs by eQTL RNA overlap for each tissue

Tissue of eQTL associations	INPUT				OUTPUT
	Distinct eQTL SNPs	Distinct eQTL RNAs	Distinct SNP-RNA associations	Distinct diseases	Prioritized disease pairs (FDR < 5%)
Adipose subcutaneous	620	489	2400	127	1581
Artery aorta	337	200	1264	96	1198
Artery tibial	492	365	1898	125	1331
Blood	270	117	1388	77	1191
Brain	188	50	554	67	448
Breast mammary tissue	238	83	987	80	970
Cells transformed fibroblasts	541	417	1485	123	799
Colon transverse	231	95	810	80	748
Esophagus mucosa	518	398	1927	126	1462
Esophagus muscularis	560	421	1856	135	1366
Heart atrial appendage	218	55	867	71	674
Heart left ventricle	378	199	1283	104	1201
Lung	154	138	416	68	45
Muscle skeletal	551	408	2240	130	1617
Nerve tibial	748	627	2497	134	1536
Pancreas	279	126	843	85	753
Skin	806	667	2669	155	1775
Stomach	202	64	779	68	676
Thyroid	857	793	2759	145	1484
Total (union of sets)	1721	2644	8033	188	2043

### Prioritized comorbidities

From the clinical NIS13 and the NEDS13 HCUP datasets (7 and 29 million patients observed at the hospital and emergency department, respectively and involving 237 disease bundles), 3032 and 4430 significant comorbidities were prioritized (OR > 1.5; FDR_comorbidity_ < 0.05; Additional file 4) accordingly, after adjusting for age, gender, and race. Overall, the union of the two findings resulted in 5200 significant comorbidities (HCUP OR > 1.5; FDR_comorbidity_ < 0.05), of which 2346 were found at OR > 3.

### Convergent genetic mechanisms between disease-pairs are enriched among comorbid diseases

Among 5200 and 2346 comorbidities respectively observed in the HCUP clinical datasets at OR > 1.5 and OR > 3 (FDR_comorbidity_ < 0.05; **Results- Disease pairs with convergent eQTL-mechanisms of genetic polymorphisms**), 398 (Additional file 1) and 211 were also predicted as sharing common mechanisms among 2043 distinct disease pairs resulted with significant eQTL RNA overlap (FDR_eRNA_ < 0.05; **Results - Disease pairs with convergent eQTL-mechanisms of genetic polymorphisms**). Thus, the enrichment of clinical comorbidities with convergent genetics resulted significant (OR = 1.6; *p*-value = 2 × 10^− 9^; Fig. 2). Moreover, the enrichment of convergent downstream eQTL RNA (overlap) reaches an odds ratio as high as 8.6 (*p*-value = 6.4 × 10^− 5^ FET) when eQTL RNA overlap is FDR_eRNA_ < 10^− 18^, and comorbidity OR > 3 and FDR_comorbidity_ < 10^− 11^. The removal of highly-related diseases using information theoretic similarity in SNOMED yielded similar results (result not shown) confirming the robustness of the approach. These results suggest that convergent eQTL regulation by distinct genetic variants may contribute in part to comorbid syndromes.

### Visualization of comorbidities sharing intergenic genetic risks through eQTL RNA overlap

The network of disease pairs prioritized as both (i) sharing eQTL RNAs significantly through their respective disease-associated eQTLs (**Methods- Statistical overlap of eQTL-associated RNAs between distinct disease-associated SNPs**) and (ii) comorbid in HCUP datasets (**Methods- Calculation of disease comorbidity based on HCUP**) is shown in Fig. 3. Two hundred and eleven disease-pairs were observed under these conservative criteria, most of which have been prioritized by eQTL associations derived from multiple relevant tissues, with each being analyzed independently (shown by the thickness of the edges). The network forms several clusters, corresponding to major disease classes (encoded by colors) such as immune-mediated diseases (the largest one), neurological diseases, cancers, metabolic diseases, cardiovascular diseases, among others. Specific examples of the patterns observed are further discussed in the caption of Fig. 3.

### Curated literature review of the prioritized eQTL-driven comorbidity network

We curated the 211 prioritized comorbidities with eQTL underpinning (Fig. 3) along with 31 (about 15% of the comorbidities) random pairs of diseases added to blind the curators (**Methods- Curation of prioritized comorbidities**). The curation results are shown in Fig. 4. Compared to controls (random set among non-prioritized disease pairs), truly prioritized disease comorbidities were significantly enriched in validated positive comorbidities in the literature (Levels 1 or 2), while controlled pairs were significantly enriched in a no-correlation curation category (Levels 5 or 6) (Chi-square test; *p* = 0.001). *The results indicate among our prioritized disease pairs, molecular mechanisms are proposed for ~ 42% of disease-pairs with known comorbidity (Levels 1 or 2) and ~ 30% for novel disease pairs (Level 5). The latter predictions had eluded clinicians and population health specialists and unveil novel clinical syndromes as well as opportunities for new therapies.*

### Case studies of biologically convergent disease comorbidities

The majority of GWAS variants in complex diseases are thought to manifest their effect through regulation [42], but the majority of identified variants lie far from genes or transcription start sites making candidate molecules and functions difficult to identify or study. The evidence from ENCODE suggests that as much as 80% of the non-coding intergenic regions are biologically interacting [21]. Figure 5 illustrates two cases of reproducible comorbidities in clinical datasets for which the shared molecular underpinning had not been identified in the literature, indicating this provides testable novel mechanisms. The networks were built as described in **Methods**- **Network visualization of the comorbidities sharing intergenic genetic risks through eQTL RNA overlap**.

Figure 5a shows the psoriasis and polycystic ovary syndrome disease pair resulted as clinically comorbid (bivariate logistic regression OR = 3.3, 95% CI: 2.8–3.9 in NIS13; OR = 68.5, 95% CI: 63.2–74.3 in NEDS13). Their independent disease-associated variants are both located in chromosome 12 but have negligible linkage disequilibrium (LD r^2^ ≤ 0.05 for any pairs of SNPs shown in the panel). rs12580100 associated with psoriasis and rs705702 with polycystic ovary syndrome are both associated by eQTL studies with the expression change of the eQTL RNA AC004057.1 on chromosome 4, a transcribed processed pseudogene, whose functions have yet to be characterized. Another example, in Fig. 5b, illustrates the prioritized molecular mechanisms between Parkinson’s disease and schizophrenia, which also are reproducibly comorbid (bivariate logistic regression OR = 4.2, 95% CI: 4.0–4.3 in NIS13; OR = 4.3, 95% CI: 4.1–4.5 in NEDS13). Immune-mediated mechanisms are being prioritized by eQTL associations as common between intergenic SNPs of these diseases, with 14 common downstream RNAs associated by eQTL with their respective distinct variants in chromosome 6. The two diseases share a cis-regulated gene IGSF9B which may inhibit synapse development [43]. Besides, two SNPs, rs17115100 and rs11191419 (LD *R*^2^ = 0.15), are associated with the expression of CYP17A1-AS1, a ncRNA within a P450 enzyme protein CYP17A1, which catalyzes many reactions and synthesis of cholesterol, steroids, and other lipids. In addition, they also share a microRNA MIR1307 [43], which further regulates a series of downstream genes. The prioritized mechanisms also recapitulate many known HLA mechanisms, thus warranting further experimental validation of the previously stated novel ones.

## Discussion

In summary, we computationally integrated and mined two clinical datasets jointly with GWAS/GTEx datasets and identified hundreds of comorbid diseases that also presented associations with convergent eQTL regulation, which may contribute in part to their common progression and pathophysiology. The substantial enrichment of convergent genetic mechanisms among comorbid diseases provides an internal validation to the methodology. The curation against the literature provides an external validation, controlled by non-prioritized diseases pairs submitted in a blinded way to curators. In addition, the disease association network recapitulated many known clinical syndromes, such as the metabolic syndrome, and identified published comorbidities for which the underpinning genetic mechanisms have yet to be unveiled. We are pursuing a systematic curation of the mechanisms results to identify known vs. novel molecular underpinnings of comorbidities. Associating diseases by their common cis-eQTL downstream mechanisms has been recently reported [16], however, their predictions have not been confirmed in clinical datasets. In addition, Hauberg et al. did not investigate trans-eQTL associations as we did in this study [16]. Altogether, such studies may provide novel mechanisms of comorbidities and provide insight for disease prevention or new therapeutic interventions.

Future studies will extend predictions beyond exact eQTL RNA overlap to use broader shared pathway memberships for distinct RNAs [19, 20, 44]. Other types of biological data (e.g., epigenetic assays and chromatin interactions in ENCODE) and clinical data (e.g., UK Biobank) could further contribute distinct and perhaps, a more accurate biological roadmap for disease comorbidity. Future studies should also focus on novel clinical syndromes that share genetic underpinning, which we are currently curating the results to identify those as well. Further, we are planning to build a publicly-accessible database with the resulting comorbid disease pairs having convergent molecular mechanisms, the network results, and an interactive visualization tool for the resulting network.

## Conclusions

This proof-of-concept study highlights the promise of integrating multiscale genomics datasets to unveil the shared molecular mechanisms of disease comorbidities. We first integrated GWAS studies with eQTL associations to discover diseases showing significantly convergent mechanisms. Then, the parallel computation of disease comorbidity using clinical datasets enabled the identification of relationships between convergent mechanisms and disease comorbidities. This subset of comorbid diseases, with convergent eQTL genetic mechanisms underpinning them, highly suggests the novel or established clinical syndromes. While it took over a decade to confirm the genetic underpinning of the metabolic syndrome, this study is likely highlighting hundreds of new ones. In addition, this knowledge could potentially improve the clinical management of comorbid syndromes with precision (using SNPs that interact), as well as shed light on novel approaches of drug repositioning, or SNP-guided precision molecular therapy even when risk variants are intergenic.

## Additional files

Additional file 1: **File S1.** Comorbid disease pairs (OR > 1.5) sharing molecular mechanisms. In the table we show all the disease pairs resulting comorbid (OR > 1.5 and FDR_comorbidity_ < 0.05) and with significant convergent mechanisms (FDR_eRNA_ < 0.05). *Disease1* and *disease2* columns represent the disease pair. The related FDRs obtained from the clinical and the molecular datasets are reported in *FDR*_*comorbidity*_ and *FDR*_*eRNA*_ columns, respectively. We reported also the resulting ORs and FDRs for both the HCUP datasets separated, i.e. NIS13 and NEDS13. For each single dataset there are two OR since they correspond to the beta related to the diseases and we derived two directional FDRs (see Methods). (XLSX 103 kb)

Additional file 2: **File S2.** Mapping between disease bundles and diseases from the GWAS Catalog. (TXT 19 kb)

Additional file 3 **File S3.** Mapping between disease bundles and ICD-9-CM diseases from the HCUP clinical datasets. (TXT 24 kb)

Additional file 4: **Figure S4.** Reproducibility of comorbidity odds ratios observed in NIS13 (hospitalizations) and NEDS13 (emergency departments) HCUP datasets. The correlation R^2^ is 0.62 and 0.63 respectively. Top disease comorbidity was measured and compared directionally, and odds ratios are shown in a log scale. (DOCX 54 kb)

## Abbreviations

CUI: Concept unique identifiers
DAG: Directed acyclic graph
dbSNP: Single nucleotide polymorphism database
EFO: Experimental factor ontology
EHR: Electronic health records
eQTL: expression Quantitative Trait Loci
eQTL RNA: eQTL-associated RNAs
FDR: False discovery rate
GTEx: Gene-Tissue expression
GWAS: Genome-Wide Association Study
HCUP: Healthcare Cost and Utilization Project
LD: Linkage disequilibrium
NEDS13: The HCUP Nationwide Emergency Department Sample
NIS13: HCUP National Inpatient Sample
OR: Odds ratio
SNOMED-CT: Systematized Nomenclature of Medicine-Clinical Terms
SNP: Single nucleotide polymorphism
UMLS: Unified medical language system
