# Supplementary material for: Novel disease syndromes unveiled by integrative multiscale network analysis of diseases sharing molecular effectors and comorbidities
Source: BMC Med Genomics. 2018 Dec 31;11(Suppl 6):112. doi: 10.1186/s12920-018-0428-9 (PMC6311938; doi:10.1186/s12920-018-0428-9)
Supplement: Supplementary file 4 — Figure S4. Reproducibility of comorbidity odds ratios observed in NIS13 (hospitalizations) and NEDS13 (emergency departments) HCUP datasets. The correlation R2 is 0.62 and 0.63 respectively. Top disease comorbidity was measured and compared directionally, and odds ratios are shown in a log scale. (DOCX 54 kb) [file 12920_2018_428_MOESM4_ESM.docx]

| 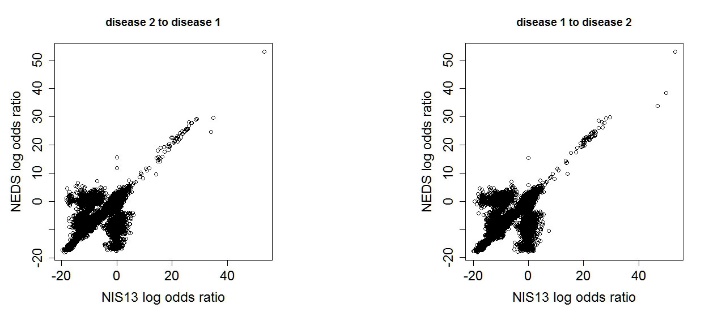 |
| --- |
| **Figure S1. Reproducibility of comorbidity odds ratios observed in NIS13 (hospitalizations) and NEDS13 (emergency departments) HCUP datasets.** The correlation R^2^ is 0.62 and 0.63 respectively. Top disease comorbidity was measured and compared directionally, and odds ratios are shown in a log scale. |
